# Deep learning-based arterial waveform analysis for predicting postoperative cerebrovascular events in pediatric patients with Moyamoya disease

**DOI:** 10.1371/journal.pone.0350637

**Published:** 2026-06-04

**Authors:** Jung-Bin Park, Youmin Shin, Jihun Kim, Yoon Jung Kim, Seung-Bo Lee, Eun-Hee Kim, Joo Whan Kim, Seung-Ki Kim, Hee-Soo Kim, Young-Gon Kim

**Affiliations:** 1 Department of Anesthesiology and Pain Medicine, Seoul National University Hospital, College of Medicine, Seoul National University, Republic of Korea; 2 Department of Transdisciplinary Medicine, Seoul National University Hospital, Republic of Korea; 3 Interdisciplinary Program in Bio-engineering, Seoul National University, Republic of Korea; 4 Department of Applied Bio-engineering, Seoul National University, Republic of Korea; 5 Department of Medical Informatics, Keimyung University School of Medicine, Republic of Korea; 6 Department of Neurosurgery, Seoul National University Hospital, College of Medicine, Seoul National University, Republic of Korea; 7 Interdisciplinary Program in Artificial Intelligence, Seoul National University, Republic of Korea; 8 Department of Medicine, College of Medicine, Seoul National University, Republic of Korea; 9 Innovative Medical Technology Research Institute, Seoul National University Hospital, Seoul, Republic of Korea; Coventry University, UNITED KINGDOM OF GREAT BRITAIN AND NORTHERN IRELAND

## Abstract

**Background:**

Postoperative cerebrovascular events, including transient ischemic attacks, infarctions, and hemorrhages, remain a significant concern in pediatric patients with Moyamoya disease (MMD)undergoing surgical revascularization. This study aimed to develop an explainable deep learning-based classification model using intraoperative arterial blood pressure (ABP) waveform analysis for postoperative cerebrovascular events in pediatric patients undergoing surgery for MMD, with exploratory analysis of associated waveform-derived physiologic features.

**Methods:**

This retrospective study included 181 pediatric patients (≤18 years) who underwent revascularization surgery for MMD, with an independent temporal holdout cohort of 79 patients reserved for validation. ABP signals were preprocessed using detrending, pulse segmentation, and normalization, then converted into image representations for deep learning classification. Various convolutional neural network (CNN) models, including ResNet50, ResNet34, DenseNet121, VGG16, and VGG19, were evaluated against Vision Transformer (ViT) architectures. Multiple image transformation methods were tested, and Grad-CAM analysis and statistical comparisons of waveform-derived physiologic features were conducted between patients with and without postoperative cerebrovascular events.

**Results:**

The optimal model configuration achieved the best performance using raw pulse waveforms with three consecutive pulses per image. CNN-based models outperformed ViT-based models, with the highest internal classification performance observed using raw pulse waveforms (AUROC = 0.772, SD = 0.070).In the independent temporal validation cohort, the model achieved an AUROC of 0.738 ± 0.011 at the patient level. Grad-CAM visualization highlighted the diastolic runoff phase as a region of interest for classification. Four waveform-derived features related to arterial compliance were significantly associated with postoperative cerebrovascular events (p < 0.05).

**Conclusions:**

In this study, CNN-based deep learning models demonstrated the feasibility of predicting postoperative cerebrovascular events from intraoperative ABP waveforms, with diastolic runoff dynamics emerging as a potentially relevant physiologic pattern. These findings are exploratory and require prospective multi-center validation before clinical application.

## Introduction

Moyamoya disease (MMD) is a rare, progressive cerebrovascular disorder characterized by stenosis or occlusion of the distal segments of the intracranial internal carotid arteries or their branches, resulting in cerebral ischemia, infarction, and neurologic deficit [1–4].

Pediatric patients with MMD remain at high risk for postoperative cerebrovascular events such as transient ischemic attacks (TIAs; up to 42.9%), cerebral infarctions (9.1–10%), and hemorrhage [5,6].These events frequently necessitate intensive clinical management, including fluid resuscitation, close hemodynamic monitoring, laboratory evaluation, and neuroimaging, and are associated with prolonged hospital stay and risk of irreversible neurologic injury. Moreover, as routine postoperative neuroimaging is not always performed, subclinical ischemic injury may go undetected. Collectively, these events highlight the need for reliable intraoperative predictors of postoperative cerebrovascular risk. However, such predictors remain limited [7,8].

Arterial blood pressure (ABP) waveform analysis provides a dynamic assessment of vascular compliance and systemic hemodynamics and may reflect physiologic characteristics relevant to cerebrovascular perfusion [9–11]. Given that MMD is often associated with systemic vasculopathy and impaired cerebrovascular reserve, intraoperative ABP waveform morphology may contain physiologic information relevant to postoperative cerebrovascular risk [3,4]. However, this potential role has not been investigated in pediatric patients with MMD. Recent advances in deep learning have enabled automated extraction of complex patterns from raw physiologic waveform data without predefined feature engineering [12,13].

This study aimed to develop an explainable deep learning-based classification model using intraoperative ABP waveform analysis and to explore waveform-derived physiologic features associated with postoperative cerebrovascular events in pediatric patients undergoing surgery for MMD.

## Materials and methods

### Data sources

This retrospective study received approval from the Institutional Review Board of Seoul National University Hospital (approval No. H-2408-031-1558, approval date: August 9, 2024, Chairperson: Hyun-Hoon Jung). An amendment for the temporal validation cohort received expedited IRB approval on April 3, 2026. The requirement for written informed patient consent was waived by the Institutional Review Board of Seoul National University Hospital owing to the retrospective nature of the study. The patient data were anonymized prior to analysis. All methods were performed in accordance with the relevant guidelines and regulations.

### Datasets

**Fig 1:**
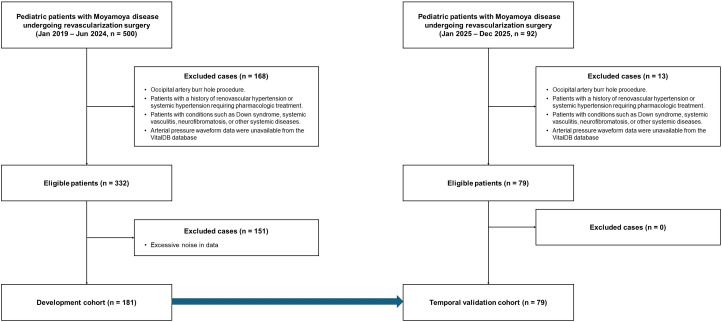
Flow chart presenting patient selection and analysis.

A total of 500 surgical cases involving pediatric patients aged ≤18 years, who underwent elective indirect revascularization surgery under general anesthesia between January 2019 and June 2024 at a tertiary referral center, Seoul National University Children’s Hospital, South Korea, were initially considered for this study. Patients were excluded if they had undergone only an occipital artery burr hole procedure, had a history of renovascular hypertension or systemic hypertension requiring pharmacologic treatment, had associated conditions such as Down syndrome, systemic vasculitis, or neurofibromatosis, or had other systemic diseases that could affect hemodynamic stability. Additionally, cases were excluded if arterial pressure waveform data were unavailable or contained excessive noise in the VitalDB database [14]. After applying these exclusion criteria, a total of 181 cases were included in the final analysis (**Fig 1**).For independent temporal validation, an additional cohort of 79 pediatric patients who underwent revascularization surgery between January 2025 and December 2025 at the same institution was separately collected using identical inclusion and exclusion criteria. This temporal hold-out cohort was entirely independent of the 181-patient development cohort and was not used at any stage of model development, being reserved solely for final validation.

We reviewed the medical records of all included patients to assess overall clinical outcomes, cerebrovascular events, and clinical status (accessed August 11, 2024). Demographic data, including age, sex, height, and weight were collected. Preoperative and postoperative imaging studies, including magnetic resonance imaging (MRI) with perfusion imaging, computed tomography (CT), and angiography, were analyzed. Intraoperative arterial pressure waveform data were obtained using the Vital Recorder Program (available at https://vitaldb.net; accessed August 10, 2024) [14]. The authors had access to identifiable patient information during the initial data collection phase, but all data were anonymized prior to analysis.

Postoperative cerebrovascular events were defined as follows: (1) the occurrence of TIAs as assessed by a neurosurgeon during the postoperative hospital stay, (2) documentation of postoperative infarctions or hemorrhages in the electronic medical records by a neurosurgeon, or (3) radiological evidence of hemorrhage or infarction identified on postoperative CT or MRI scans. We included TIAs as part of the composite outcome, as postoperative TIAs in this population frequently require diagnostic evaluation, medical optimization, or hemodynamic management, and therefore represent clinically meaningful events despite their transient nature.

### Data preprocessing

The ABP signals were converted into digital form at a sampling frequency of 100 Hz. Clean segments of at least 10 minutes were extracted through visual inspection to ensure signal quality. Several preprocessing steps were performed to refine the signals:

1. Detrending: A detrending process was applied to remove low-frequency drifts and baseline fluctuations in the ABP signals using a 0.5 Hz band-pass filter.
2. Pulse separation: To segment individual pulses within the ABP waveform, we employed two complementary methods: a rule-based approach and PyPPG function-based detection [15]. The rule-based method identified systolic peaks as local maxima and determined pulse onset by detecting the minimum value within 30 sample points preceding each peak, while the pulse offset was defined as the subsequent trough. In parallel, the PyPPG function provided additional onset and offset estimates, enhancing segmentation accuracy through signal processing techniques optimized for pulse wave analysis. In cases where the two methods produced discrepant results, visual inspection was performed to ensure accurate segmentation, though such instances were rare. By integrating both approaches, we achieved robust and precise pulse identification, minimizing segmentation errors.
**3.** Normalization: To minimize inter-patient variability, each sample was constructed by grouping three consecutive pulses into a single unit. The Y-axis was normalized using min-max scaling (0–1) to standardize amplitude, while the X-axis was resized to a fixed length of 300 data sample points to ensure uniform temporal representation.

### Model development

To classify postoperative cerebrovascular events, we employed two categories of deep learning models: CNN-based architectures and ViT-based architectures [16–18]. The CNN-based models included ResNet50, ResNet34, DenseNet121, VGG16, and VGG19, while the ViT-based models comprised ViT-Small, ViT-Base, ViT-Large, and ViT-Base with CLIP pre-trained [19–21]. However, ViT-based models failed to learn meaningful representations and showed no convergence during training. Due to their poor performance, they were excluded from further experiments.

Various methods were considered for converting ABP signals into image representations, including Gramian Angular Summation Field (GASF), Markov Transition Field (MTF), Recurrence Plot (RP), Spectrograms (SPEC), and direct raw pulse plotting (DRP) (**S1 Fig in S1 File**) [22–25]. Among these, the raw pulse waveform plot demonstrated the best classification performance. Consequently, all model training and evaluation were conducted using raw pulse image representations.

The 181-patient development cohort was split at the patient level into training, validation, and internal test sets (70%/15%/15%), repeated five times using different random seeds. All splits were performed strictly at the patient level to prevent waveform instances from the same patient from being shared across sets. The independently collected temporal hold-out cohort (n = 79, January 2025–December 2025) was not used at any stage of model development and was reserved solely for final validation. The models were trained using the Adam optimizer with a learning rate of 0.001, and early stopping was employed based on validation loss with a patience of 10 epochs to mitigate overfitting. Data augmentation (random horizontal flipping, rotation, and color jittering) was applied to improve generalization performance.

For benchmarking purposes, handcrafted waveform features were also used to train conventional machine learning (ML)models, including logistic regression, random forest, support vector machine, and k-nearest neighbors. To further improve baseline performance, feature selection techniques (Recursive feature elimination, F-value ranking, and mutual information scoring) were applied prior to model training. These models served as traditional ML comparators to the deep learning classifier (**S1 Table in S1 File**).

### Instance Aggregation Analysis

To determine the optimal number of pulses per image and the most effective aggregation strategy, we conducted additional analyses exploring different approaches to image construction and classification decision-making.

First, we investigated the impact of the number of pulses included in a single image, assessing how varying pulse counts influenced classification performance. Next, we evaluated voting-based ensemble methods, analyzing the optimal number of instances to aggregate for a more robust classification decision. Lastly, we implemented a Multiple Instance Learning (MIL)-based approach, utilizing a top-k aggregation strategy, where predictions were made at the individual pulse level, and the top k highest-confidence instances were aggregated to derive a case-level decision [26].

### CAM visualization

Class Activation Mapping (CAM) was used to interpret the model’s decision-making process, with Grad-CAM applied to highlight salient regions in the input images [27]. Activation maps from the final convolutional layer initially highlighted the entire image rather than specific regions of interest. To improve localization, we analyzed the penultimate convolutional layer, which provided more focused and interpretable feature activations.

### Statistical comparison

To extract features centered on the notch point, the ABP signal was first normalized within each pulse segment, specifically from peak to offset. This normalization was applied to minimize the impact of variations between the onset and peak, which could otherwise bias statistical calculations. Following normalization, the signal was divided into three segments: peak to dicrotic notch(DN), DN to diastolic peak (DP), and DP to offset.

For each segment, features were extracted from raw signals, and their first and second derivatives, including segment length, slope, minimum and maximum values, mean, median, and area under the receiving operating curve (AUROC). Outliers were removed based on the interquartile range (IQR) to minimize biases arising from the limited sample size.

Feature distributions between patients with and without postoperative cerebrovascular events were compared using the Mann-Whitney U-test or independent t-test, depending on data normality assessed by the Shapiro-Wilk test. A significance level of p < 0.05 was set for all comparisons. Statistical analysis was performed using the Python 3.8 software.

## Results

Of the 181 cases, 125 (69.1%) experienced postoperative cerebrovascular events during hospitalization (**Table 1**). Demographic characteristics, including age, sex, height, and weight, were comparable between the two groups. The surgical approach, posterior circulation involvement, and Suzuki stage showed no significant differences. A total of 10 patients (5.52%) developed postoperative infarction, while 3 patients (1.66%) experienced postoperative hemorrhage.

**Table 1:** Baseline characteristics in the dataset.

	Total (n = 181)	Patients with postoperative cerebrovascular events (n = 125)	Patients without postoperative cerebrovascular events (n = 56)	*p*-Value
Age (years)	8.40 ± 4.44	8.39 ± 4.62	8.43 ± 4.06	0.661
Sex (M/F)	93/88 (51.38/48.62)	67/58 (53.60/46.40)	26/30 (46.43/53.57)	0.232
Height	133.64 ± 23.19	134.06 ± 22.89	132.64 ± 24.10	0.726
Weight	37.00 ± 19.53	37.82 ± 20.26	35.03 ± 17.72	0.561
Operation name				
EDAS	120 (66.30%)	81 (64.80%)	39 (69.64%)	0.320
EDAS with bifrontal EGS or multiple burr hole surgery	61 (33.70%)	44 (35.20%)	17 (30.36%)	
PCA involvement	45 (24.9%)	40 (32.00%)	17 (30.36%)	0.481
Suzuki stage				0.293
Stage 1–2	27 (14.9%)	18 (14.4%)	9 (16.1%)	
Stage 3–4	123 (68.0%)	89 (71.2%)	34 (60.7%)	
Stage 5–6	31 (17.1%)	18 (14.4%)	13 (23.2%)	
Median (IQR)	3 (3-4)	3 (3-4)	3 (3-4)	
Postoperative infarction	10 (5.52%)	10 (8.00%)	0	N/A
Postoperative hemorrhage	3 (1.66%)	3 (2.40%)	0	N/A

EDAS, encephalduroarterio-synangiosis; EGS, encephalogaleosynangiosis; IQR, interquartile range; N/A, not applicable; PCA, posterior cerebral artery.

Data are presented as mean ± standard deviations or number (percentage).

To classify postoperative cerebrovascular events, we compared various deep learning architectures using different ABP signal image representations. CNN-based models (ResNet50, ResNet34, DenseNet121, VGG16, and VGG19) consistently outperformed ViT-based models (ViT-Small, ViT-Base, ViT-Large, and ViT-Base with CLIP pre-trained). The ViT models failed to learn meaningful representations and showed poor classification performance, leading to their exclusion from further analysis (**S2 Table in S1 File**).

Among the different ABP signal-to-image conversion methods, raw pulse waveform plots demonstrated the best classification performance (**Table 2**), outperforming GASF, MTF, RP, and Spectrograms. Consequently, all final training and evaluation were conducted using raw pulse image representations.

**Table 2:** CNN-based deep learning model classification performance.

Models	Image type	AUROC image	AUROC patient
ResNet-50	GASF	0.637 ± 0.039	0.684 ± 0.024
	MTF	0.643 ± 0.031	0.678 ± 0.035
	RP	0.654 ± 0.020	0.682 ± 0.021
	SPEC	0.644 ± 0.076	0.720 ± 0.050
	**DRP**	**0.713 ± 0.051**	**0.754 ± 0.061**
DenseNet-121	GASF	0.675 ± 0.021	0.711 ± 0.010
	MTF	0.675 ± 0.039	0.702 ± 0.047
	RP	0.711 ± 0.006	0.718 ± 0.015
	SPEC	0.690 ± 0.047	0.730 ± 0.055
	DRP	0.645 ± 0.057	0.678 ± 0.061
VGG16	GASF	0.672 ± 0.056	0.708 ± 0.046
	MTF	0.588 ± 0.053	0.609 ± 0.048
	RP	0.596 ± 0.031	0.612 ± 0.030
	SPEC	0.641 ± 0.110	0.675 ± 0.120
	DRP	0.621 ± 0.031	0.639 ± 0.024
VGG19	GASF	0.648 ± 0.030	0.673 ± 0.039
	MTF	0.596 ± 0.045	0.635 ± 0.030
	RP	0.652 ± 0.030	0.667 ± 0.017
	SPEC	0.669 ± 0.074	0.709 ± 0.094
	DRP	0.638 ± 0.014	0.655 ± 0.010

AUROC, Area under the receiver operating characteristic curve;CNN, Convolutional neural network; DRP, Direct raw pulse;GASF, Gramian angular summation field; MTF, Markov transition field; RP, Recurrence plot; SPEC, Spectrogram; ViT, Visual transformer.

**Table 3** presents the impact of the number of pulses per image and the number of instances used for aggregation in classification. Performance initially improved as the number of pulses per image increased, but plateaued or slightly declined beyond a certain threshold. A voting-based ensemble strategy was applied, and the optimal number of instances for aggregation was determined, contributing to improved classification stability. Additionally, a MIL approach with top-k aggregation was implemented, where classification decisions were derived from the highest-confidence instances. Although MIL achieved reasonable performance (AUROC = 0.740, SD (standard deviation) = 0.011), it did not yield the best results, suggesting that although it provided robustness, it was not the optimal aggregation strategy. The optimal classification performance was achieved when a single image containing three pulses was used as input. This configuration yielded the highest AUROC (0.772) with the lowest SD (0.070). For comparison, the best-performing CNN configuration outperformed all traditional ML baselines trained on handcrafted features, suggesting a potential advantage of end-to-end representation learning over feature-engineered models (**S3 Table in S1 File**).

**Table 3:** Comparison across different number of pulses and images (ResNet-50, DRP).

Number of pulse	Number of image	AUROC image	AUROC patient
1	3	0.684 ± 0.082	0.734 ± 0.100
**3**	**3**	**0.713 ± 0.051**	**0.754 ± 0.061**
5	3	0.696 ± 0.098	0.725 ± 0.088
7	3	0.642 ± 0.061	0.720 ± 0.059
**3**	**1**	**0.772 ± 0.070**	**0.772 ± 0.070**
3	3	0.713 ± 0.051	0.754 ± 0.061
3	5	0.682 ± 0.056	0.731 ± 0.039
3	7	0.710 ± 0.047	0.756 ± 0.050
3	9	0.652 ± 0.068	0.707 ± 0.084
MIL		–	0.740 ± 0.011

AUROC, Area under the receiver operating characteristic curve; DRP, Direct raw pulse; MIL, Multiple instance learning.

The final model configuration was additionally evaluated in an independent temporal hold-out cohort (n = 79). Baseline characteristics of the temporal hold-out cohort were generally comparable to those of the development cohort (**S4 Table in S1 File**). The model achieved an AUROC of 0.712 ± 0.022 at the image level and 0.738 ± 0.011 at the patient level. Threshold-based classification metrics and calibration results for both the internal test set and the temporal hold-out cohort are summarized in **S5 Table in S1 File**. Compared with the ML and MIL models, the best-performing deep learning model showed higher sensitivity in both cohorts, although this was accompanied by lower specificity in the temporal hold-out cohort. However, the performance differences between the best-performing deep learning model and the comparator models were not statistically significant in the internal evaluation (DL vs. ML, p = 0.380 ± 0.109; DL vs. MIL, p = 0.495 ± 0.188)

**Fig 2:**
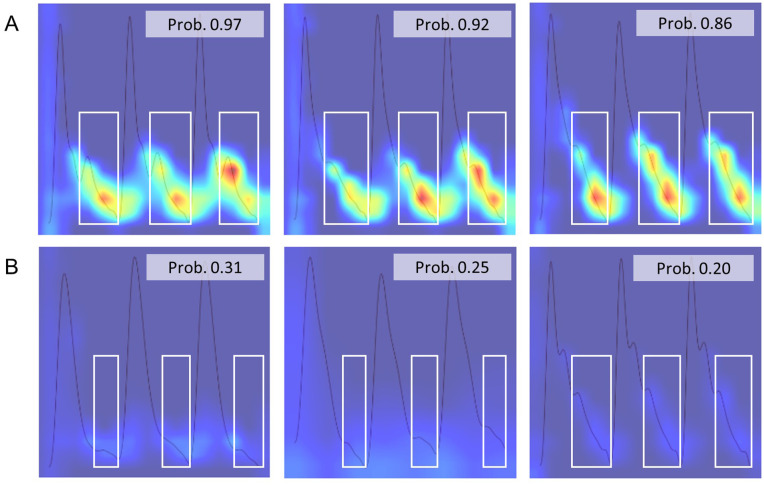
Grad-CAM visualization of the CNN model highlighting the diastolic runoff region. Representative Grad-CAM activation maps are shown for a case with postoperative cerebrovascular events (A) and a case without events **(B)**. In the case with postoperative cerebrovascular events, the model showed stronger activation over the diastolic phase, particularly the diastolic runoff region (white box), suggesting that this waveform segment may be relevant to the model’s prediction. The prediction output for each case is shown together with the corresponding Grad-CAM overlay.

The final convolutional layer exhibited a tendency to highlight the entire image rather than focusing on specific regions of interest. To obtain more localized feature activations, the preceding convolutional layer was examined (S2 Fig in S1 File). As shown in Fig 2, Grad-CAM visualization revealed that in patients with postoperative cerebrovascular events, the diastolic runoff region was distinctly highlighted, suggesting that this region may be relevant for classification.

**Fig 3:**
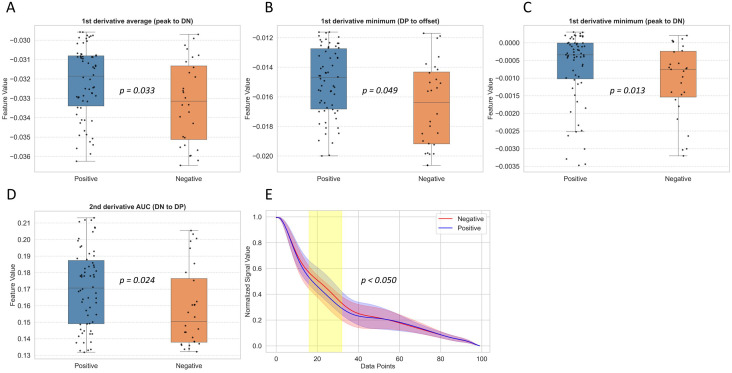
Statistical comparison of waveform-derived features between patients with and without postoperative cerebrovascular events. Panels A–D show the distribution of the four physiologic features that were significantly different between the two groups (p < 0.05). Panel E presents a point-by-point comparison across the diastolic segment of the waveform, illustrating localized regions where the patients with postoperative cerebrovascular events exhibited steeper decay in pressure.

After excluding patient cases that exhibited outliers in specific features, a time point-by-point statistical comparison between the patients with postoperative cerebrovascular events (event group) and those without (non-event group) was conducted (**Fig 3**). The visually apparent differences observed between the two groups were also statistically validated. Further analysis revealed that four features demonstrated statistically significant differences (U-test, p < 0.05), all of which were significantly higher in the event group. These features included the mean of the first derivative from peak to DN, the minimum value of the first derivative from DN to DP, the minimum value of the first derivative from DP to offset, and the AUROC of the second derivative from peak to DN.

## Discussion

This study aimed to develop a deep learning-based model and explore whether intraoperative ABP waveform analysis could identify physiologic signatures associated with postoperative cerebrovascular events in pediatric patients undergoing revascularization surgery. Our findings showed that CNN-based models outperformed ViT-based models in classifying postoperative cerebrovascular events. Among various ABP signal-to-image transformations, raw waveform images showed the best performance, with three pulses per image yielding an AUROC of 0.772. Grad-CAM analysis highlighted the diastolic runoff region as a discriminative region, and four waveform-derived features were significantly associated with postoperative cerebrovascular events. To our knowledge, this is the first study to apply deep learning to intraoperative ABP waveform analysis for predicting postoperative cerebrovascular events in pediatric patients with MMD.

CNN-based models demonstrated robust classification performance, whereas ViT-based models failed to achieve stable convergence. One possible explanation is that, unlike natural images where pixel-wise relationships and global textures provide meaningful information, ABP waveforms exhibit distinct morphological structures, such as systolic upstroke, dicrotic notch, and diastolic downstroke, which may not be effectively captured by ViT’s self-attention mechanism alone [9,28]. In contrast, CNNs are designed for local feature extraction and hierarchical pattern recognition, which may be advantageous for capturing subtle variations in waveform morphology [28]. In medical signal analysis, CNN-based architectures have shown favorable performance compared with transformer-based models for tasks requiring structured feature extraction from physiologic waveforms [29,30]. Prior studies in electrocardiography and electroencephalography analysis have reported similar findings, particularly with raw signals [31,32]. ViT performance may be limited in settings with limited data, as transformers typically require large-scale datasets to learn generalizable features [17,18]. Additionally, class imbalance may have further contributed to ViT’s difficulty in learning discriminative representations in this relatively small dataset [28].

While alternative image transformation methods, such as GASF, MTF, RP, and spectrograms, offer different perspectives for representing the signal, raw pulse plots yielded the highest classification performance. This may reflect the relatively constrained feature space of ABP waveforms, where complex transformations do not necessarily enhance discriminability. Regarding input construction, a single image containing three consecutive pulses outperformed configurations using multiple images per case, suggesting that increasing the number of images may introduce redundancy rather than additional informative variation. These findings suggest the importance of careful input selection in waveform-based deep learning, as simpler representations may provide more stable inputs in settings with limited data.

Grad-CAM analysis revealed relatively stronger activation in the diastolic phase, which was consistent with four statistically significant derivative-based waveform features. These derivative features may reflect subtle variations in ABP waveform morphology that may be associated with differences in vascular compliance or flow dynamics among patients who develop postoperative cerebrovascular events. For example, the elevated mean of the first derivative from peak to DN suggests a steeper rise in ABP, whereas reduced minimum values of the first derivative in the DN-to-DP and DP-to-offset intervals indicate a more abrupt decline in ABP. The increased AUROC of the second derivative from DN to DP was also consistent with this pattern. A rapid diastolic runoff has been associated with decreased vascular resistance, hypovolemia, or impaired blood flow compensation, potentially leading to insufficient distal perfusion [10,33]. These observations raise the possibility that diastolic-phase waveform alterations may reflect impaired maintenance of continuous forward flow during diastole, potentially influencing cerebral blood flow dynamics.

In patients with MMD, impaired diastolic hemodynamics are physiologically relevant because progressive stenosis of the distal internal carotid and proximal cerebral arteries reduces cerebrovascular reserve and limits compensatory blood flow capacity [3]. The diastolic waveform pattern observed in the event group resembles patterns reported in aging populations, where reduced diastolic flow has been associated with increased vulnerability to hypoperfusion [34]. Although the underlying mechanisms differ, this observation suggests a potential link between diastolic flow characteristics and susceptibility to cerebral hypoperfusion in MMD. However, this interpretation remains speculative and requires prospective validation.

From an intraoperative perspective, these findings may indicate that diastolic hemodynamic stability is a relevant physiologic consideration in pediatric MMD patients. However, given the qualitative nature of Grad-CAM and the exploratory design of this study, these observations should be regarded as exploratory rather than conclusive, and further prospective validation is required before clinical recommendations can be made.

Previous studies have shown that greater intraoperative blood pressure variability and abrupt decreases in blood pressure are independently associated with an increased risk of postoperative cerebral infarction [7]. Our findings suggest that intraoperative ABP waveforms may contain additional prognostic information beyond conventional blood pressure metrics. Furthermore, patient-specific vascular properties, as reflected in waveform-derived features, may influence postoperative outcomes. Future studies integrating multimodal physiologic data could provide deeper insights into the complex interplay between intraoperative hemodynamics, vascular pathology, and surgical outcomes inpatients with MMD.

For benchmarking purposes, we also evaluated traditional ML models trained on handcrafted waveform features (**S3 Table in S1 File**). Although the CNN-based classifier showed the best overall discrimination among the evaluated approaches, the performance gains over the comparator models were modest and not statistically significant in the current cohort. Therefore, the present findings should not be interpreted as definitive evidence of the superiority of deep learning over simpler approaches. Rather, the main value of the CNN-based model in this study lies in its ability to learn directly from raw waveform representations without handcrafted feature engineering and to provide additional interpretability through Grad-CAM, which identified the diastolic runoff region as a discriminative pattern. These findings were further supported by independent statistical analyses of waveform-derived physiologic features. Taken together, these results suggest the feasibility of deep learning as an end-to-end representation-learning framework for raw ABP waveform analysis, rather than establishing its definitive superiority as a predictive model.

To address class imbalance, we used a class-weighted loss with patient-level validation during training to mitigate model bias toward the majority class. We also tested data-level rebalancing (oversampling and undersampling), but these approaches did not improve performance. Specifically, oversampling introduced redundancy and led to mild overfitting, while undersampling reduced informative waveform variability. These findings suggest that class weighting may be a more suitable strategy for this dataset (**S6 Table in S1 File**).

The best-performing deep learning model showed relatively high sensitivity in both the internal and temporal validation cohorts, which may be clinically advantageous in the context of postoperative cerebrovascular monitoring. In pediatric MMD patients, missing a high-risk patient has greater clinical consequence than a false-positive prediction, as undetected events may result in delayed intervention and irreversible neurologic injury. From this perspective, a screening-oriented tool that prioritizes sensitivity, even at the cost of some specificity, may be appropriate in this population. However, the modest overall discrimination and the reduction in specificity observed in the temporal cohort highlight the current limitations of the model as a standalone decision-support tool. Currently, no validated clinical risk scoring system exists for predicting postoperative cerebrovascular events in pediatric MMD, and the present model should be regarded as a proof-of-concept framework rather than a deployment-ready tool. In this context, the model may provide an additional layer of physiologic insight beyond conventional hemodynamic parameters, potentially helping clinicians recognize vulnerable hemodynamic states and prompt closer monitoring or individualized hemodynamic assessment. While the current study does not define specific intervention thresholds, prospective studies evaluating waveform-guided management strategies would be a reasonable next step toward clinical translation.

Our study has several limitations. First, this was a single-center retrospective study with a limited sample size, which restricts generalizability. True external multi-center validation was not feasible, as our institution performs the majority of pediatric MMD revascularization surgeries in the country, and comparable independent datasets of sufficient quality and scale are not available elsewhere. Although we additionally performed an independent temporal validation using a chronologically separated cohort from the same institution, this does not constitute true external validation, and generalizability beyond our institution remains to be established. In addition, although all dataset splits were performed strictly at the patient level to minimize data leakage and robustness was explored through repeated seed-based evaluation, different pulse-per-image settings, varying numbers of aggregated images, and an MIL-based aggregation strategy, these measures cannot fully eliminate the risk of overfitting in a relatively small cohort. Given these constraints, the present work should be regarded as a feasibility study, and prospective multi-center external validation will be necessary before clinical application. Furthermore, because the observed performance differences between the deep learning model and comparator models were modest and not statistically significant, this study does not establish definitive superiority of deep learning, and larger-scale validation will be required to determine whether its added complexity is justified. Second, the composite outcome included TIAs alongside infarctions and hemorrhages. While this definition reflects the clinical relevance of TIAs in perioperative management of pediatric MMD, the low incidence of major cerebrovascular events precluded separate analysis of these endpoints. Future studies with larger cohorts should evaluate model performance across distinct cerebrovascular outcome categories. Third, we did not incorporate well-established clinical risk factors, such as posterior cerebral artery involvement or a history of previous neurological insults, which may influence postoperative outcomes. This study was specifically designed to determine whether intraoperative arterial waveform morphology alone provides physiologic signatures associated with postoperative cerebrovascular events. Incorporating strong clinical predictors into a relatively small and imbalanced dataset could have increased the risk of overfitting and caused the deep learning model to rely predominantly on those features, thereby obscuring the incremental contribution of waveform-derived information. Future studies with larger cohorts are needed to develop multimodal models that integrate angiographic and clinical severity with waveform-based physiologic features.

In conclusion, this study demonstrates the feasibility of predicting postoperative cerebrovascular events from intraoperative ABP waveforms using CNN-based deep learning models in pediatric MMD patients. Derivative-based waveform features, particularly diastolic runoff dynamics, may represent physiologically relevant patterns associated with postoperative cerebrovascular events. Future prospective multi-center studies are needed to validate these findings and determine the clinical utility of waveform-based risk stratification in this population.

## Supporting information

S1 File Supporting figures and tables. (DOCX)
