# Supplementary material for: Deep learning-based arterial waveform analysis for predicting postoperative cerebrovascular events in pediatric patients with Moyamoya disease
Source: PLoS One. 2026 Jun 4;21(6):e0350637. doi: 10.1371/journal.pone.0350637 (PMC13235888; doi:10.1371/journal.pone.0350637)
Supplement: S1 File — (DOCX) [file pone.0350637.s001.docx]

**S1 Fig. Various images generated by signal** A: Gramian angular summation field, B: Markov transition field, C: Recurrence Plot, D: Spectrograms, and E: Direct raw pulse plot.

**
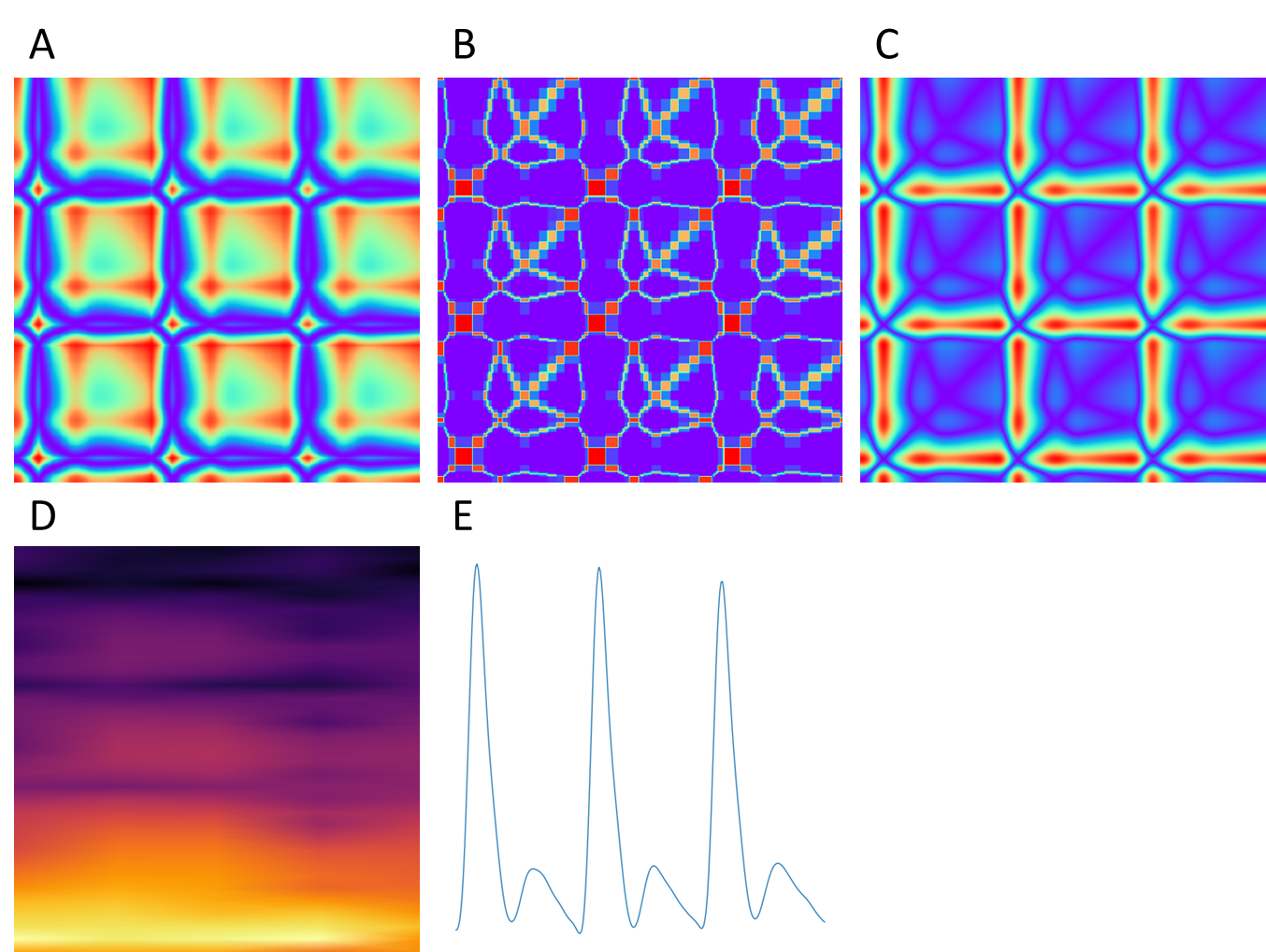
**

**S2 Fig. Grad-CAM analysis for the conv3.3 layer.** Representative Grad-CAM activation maps from the conv3.3 layer are shown for true-positive cases (A) and true-negative cases (B). In true-positive cases, the conv3.3 layer showed broad and relatively diffuse activation across the waveform, rather than localized activation confined to a specific waveform segment. These findings suggest that the conv3.3 layer mainly captured global class-discriminative information but provided limited temporal specificity for identifying clinically interpretable waveform regions.
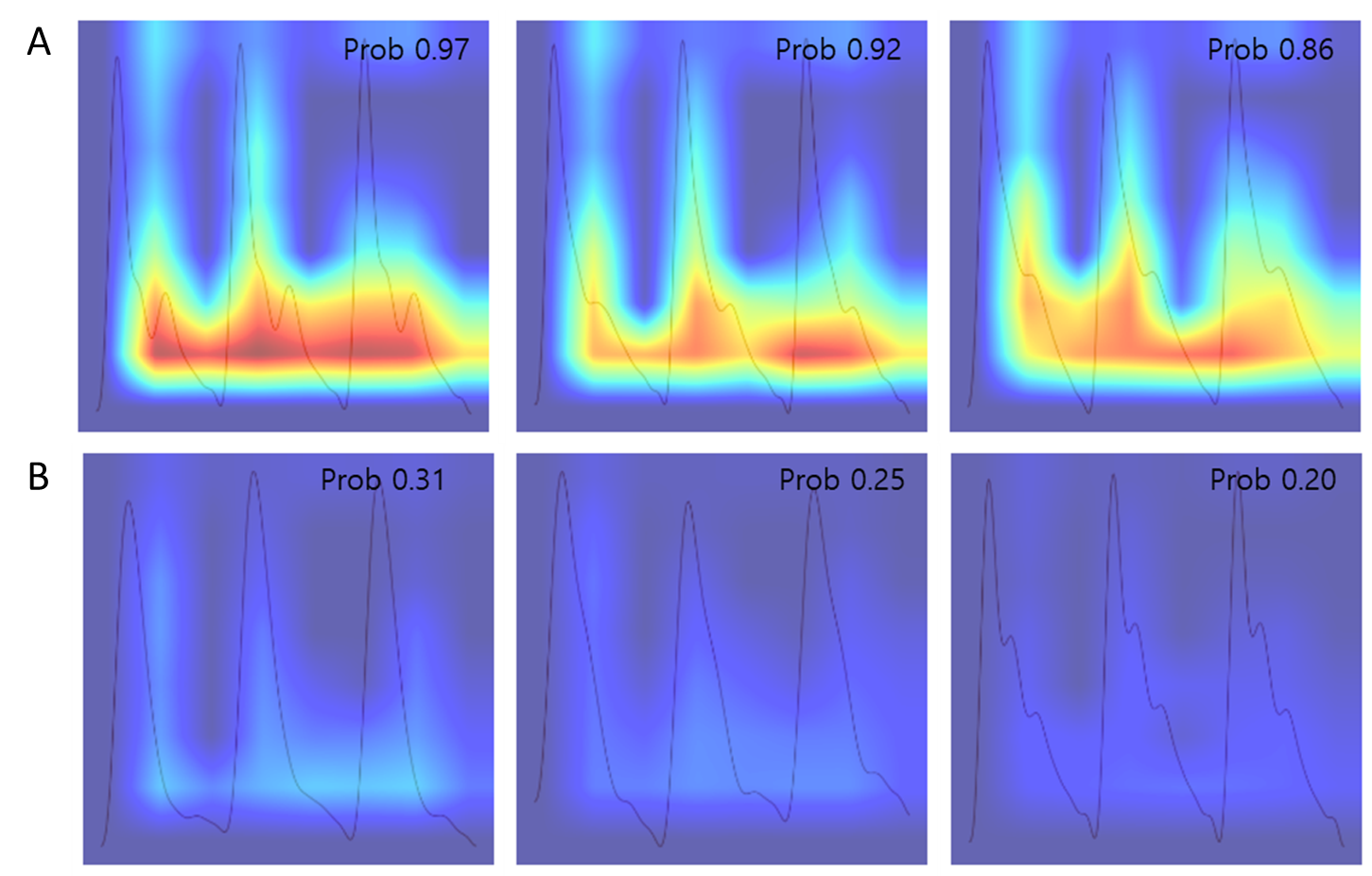


**S1 Table. Categories of handcrafted physiologic features extracted from intraoperative arterial blood pressure waveforms (Total 125 features).**

| Category | Description | Physiologic relevance | Example feature names |
| --- | --- | --- | --- |
| Morphology  (raw pulse shape) | Basic geometric characteristics of each pulse | Reflects forward pulse propagation, arterial stiffness, and upstroke velocity from onset to peak | length_of_sig_mean; max_sig_mean; height_sig_mean; gradient_from_start_mean |
| Morphology  (secondary peaks) | Number and prominence of intra-pulse hills | Surrogate for wave reflection / vessel elasticity | num_hills_mean; num_hill_thres3_mean; num_hill_thres5_mean |
| Slope & Decay Dynamics | Steepness and variability in decline phase | Reflects diastolic runoff / distal perfusion resistance | steepest_gradient_mean; mean_gradient; std_gradient |
| Statistical descriptors | Distributional properties of pulse waveform | Captures overall signal variability and asymmetry | mean_value; median_value; std_dev; variance; sig_skewness; sig_kurtosis |
| Energy-based metrics | Total signal power | Associated with waveform volatility / hemodynamic load | sig_energy |
| Spectral entropy | Spectral dispersion of waveform | Reflects irregularity / instability in vascular tone | sig_entropy |
| Frequency-domain | Frequency-specific magnitude and phase components | Captures oscillatory characteristics & vascular compliance | Magnitude_1-50; Phase_1-50 |
| RR interval surrogates  (HRV-like) | Beat-to-beat variability features | Captures beat-to-beat (pulse-to-pulse) variability in epoch length as a surrogate of autonomic and hemodynamic regulation | sdnn; rmssd; pnn50; hf; lf; sd1; sd2 |

HRV, Heart Rate Variability; RR, R-R interval; SDNN, Standard Deviation of NN intervals; RMSSD, Root Mean Square of Successive Differences; PNN50, Percentage of NN intervals differing by >50 ms; HF, High-Frequency power; LF, Low-Frequency power; SD1, Poincaré plot short-axis standard deviation; SD2, Poincaré plot long-axis standard deviation; sig, Signal; num_hills_thres3_mean, mean number of intra-pulse hills exceeding 30% of peak amplitude.

**S2 Table. ViT-based deep learning model classification performance**

| **Models** | **Image type** | **AUROC image** | **AUROC patient** |
| --- | --- | --- | --- |
| ViT small | GASF | 0.503 ± 0.082 | 0.498 ± 0.093 |
| ViT small | MTF | 0.545 ± 0.086 | 0.580 ± 0.085 |
| ViT small | RP | 0.614 ± 0.068 | 0.622 ± 0.076 |
| ViT small | SPEC | 0.545 ± 0.099 | 0.549 ± 0.052 |
| ViT small | DRP | 0.536 ± 0.054 | 0.583 ± 0.058 |
| ViT base | GASF | 0.618 ± 0.097 | 0.605 ± 0.069 |
| ViT base | MTF | 0.528 ± 0.035 | 0.580 ± 0.063 |
| ViT base | RP | 0.685 ± 0.068 | 0.681 ± 0.041 |
| ViT base | SPEC | 0.517 ± 0.068 | 0.578 ± 0.058 |
| ViT base | DRP | 0.508 ± 0.042 | 0.539 ± 0.068 |
| ViT large | GASF | 0.582 ± 0.068 | 0.580 ± 0.098 |
| ViT large | MTF | 0.549 ± 0.081 | 0.578 ± 0.101 |
| ViT large | RP | 0.524 ± 0.019 | 0.513 ± 0.035 |
| ViT large | SPEC | 0.675 ± 0.994 | 0.731 ± 0.114 |
| ViT large | DRP | 0.588 ± 0.087 | 0.647 ± 0.098 |
| ViT clip | GASF | 0.625 ± 0.075 | 0.613 ± 0.085 |
| ViT clip | MTF | 0.624 ± 0.079 | 0.622 ± 0.087 |
| ViT clip | RP | 0.503 ± 0.081 | 0.511 ± 0.065 |
| ViT clip | SPEC | 0.635 ± 0.105 | 0.697 ± 0.097 |
| ViT clip | DRP | 0.501 ± 0.038 | 0.503 ± 0.025 |

AUROC, Area under the receiver operating characteristic curve; DRP, Direct raw pulse; GASF, Gramian angular summation field; MTF, Markov transition field; RP, Recurrence plot; SPEC, Spectrogram; ViT, Visual transformer.

**S3 Table. Comparison of baseline machine learning models using handcrafted waveform features and the proposed deep learning classifier.**

| Model type | Algorithm | Configuration | AUROC (patient-level) |
| --- | --- | --- | --- |
| Traditional ML | Logistic Regression | MI (K=18) | 0.646±0.023 |
|  | Support vector machine | F_value (K=14) | 0.623±0.010 |
|  | KNN | F_value (K=22) | 0.653±0.033 |
|  | Random Forest | RFE (K=24) | 0.684±0.019 |
| Deep Learning (proposed) | CNN (ResNet-50) | Raw waveform  (3 pulses, 1 image) | 0.772±0.070 |

ML, machine learning; KNN, k-nearest neighbors; RFE, recursive feature elimination; MI, mutual information; F-value, F-statistic–based feature selection; CNN, convolutional neural networks; AUROC, area under the receiver operating characteristic curve.

**S4 Table. Baseline characteristics of the development and temporal validation cohorts.**

|  | Development cohort (n=181) | Temporal hold-out cohort (n=79) | *p*-Value |
| --- | --- | --- | --- |
| Age (years) | 8.40 ± 4.44 | 8.70 ± 4.05 | 0.607 |
| Sex (M/F) | 93/88 (51.38/48.62) | 41/38 (51.9/48.1) | 1.000 |
| Height (cm) | 133.64 ± 23.19 | 137.77 ± 21.28 | 0.177 |
| Weight (kg) | 37.00 ± 19.53 | 39.30 ± 17.68 | 0.370 |
| Operation name |  |  | 1.000 |
| EDAS | 120 (66.3%) | 52 (65.8%) |  |
| EDAS with bifrontal EGS or multiple burr hole surgery | 61 (33.7%) | 27 (34.2%) |  |
| PCA involvement | 45 (24.9%) | 18 (22.8%) | 0.840 |
| Suzuki stage |  |  | 0.609 |
| Stage 1-2 | 27 (14.9%) | 14 (17.7%) |  |
| Stage 3-4 | 123 (68.0%) | 55 (69.6%) |  |
| Stage 5-6 | 31 (17.1%) | 10 (12.7%) |  |
| Median (IQR) | 3 (3-4) | 3 (3-4) |  |
| Postoperative cerebrovascular events | 125 (69.1%) | 52 (65.8%) | 0.711 |
| Postoperative infarction | 10 (5.52%) | 8 (10.1%) | 0.281 |
| Postoperative hemorrhage | 3 (1.66%) | 3 (3.8%) | 0.372 |

EDAS, encephalduroarteriosynangiosis; EGS, encephalogaleosynangiosis; IQR, interquartile range; PCA, posterior cerebral artery.

Data are presented as mean ± standard deviations or number (percentage).

**S5 Table**. Threshold-based classification performance and calibration results of the deep learning, machine learning, and multiple instance learning models in the internal test set and temporal validation cohort)

| ***Models*** | ***AUROC*** | ***Accuracy*** | ***Sensitivity*** | ***Specificity*** | ***Brier score*** |
| --- | --- | --- | --- | --- | --- |
| *Internal test* | | | | | |
| *DL (best)* | *0.772 ± 0.070* | *0.733 ± 0.041* | *0.825 ± 0.143* | *0.695 ± 0.114* | *0.174 ± 0.007* |
| *ML (best)* | *0.684 ± 0.019* | *0.711 ± 0.080* | *0.725 ± 0.224* | *0.705 ± 0.206* | *0.215 ± 0.004* |
| *MIL* | *0.740 ± 0.011* | *0.763 ± 0.050* | *0.675 ± 0.168* | *0.800 ± 0.036* | *0.191 ± 0.007* |
| *Temporal validation* | | | | | |
| *DL (best)* | *0.738 ± 0.011* | *0.664 ± 0.038* | *0.846 ± 0.082* | *0.573 ± 0.094* | *0.203 ± 0.003* |
| *ML (best)* | *0.669 ± 0.020* | *0.644 ± 0.060* | *0.777 ± 0.202* | *0.577 ± 0.186* | *0.228 ± 0.005* |
| *MIL* | *0.740 ± 0.094* | *0.674 ± 0.021* | *0.823 ± 0.117* | *0.600 ± 0.083* | *0.197 ± 0.005* |

AUROC, Area under the receiver operating characteristic curve; DL, Deep learning; ML, Machine learning; MIL, Multiple instance learning.

**S6 Table**. **Performance comparison of different imbalance handling strategies applied to the best-performing model configuration (ResNet 50; Number of pulse 3; Number of image 1).**

| **Balancing methods** | **AUROC image/patient level** |
| --- | --- |
| Class weight | 0.772±0.070 |
| Minority over-sampling | 0.763±0.033 |
| Majority down-sampling | 0.721±0.062 |

AUROC, Area under the receiver operating characteristic curve.
